# Effect of bacterial infection on sperm quality and DNA fragmentation in subfertile men with Leukocytospermia

**DOI:** 10.1186/s12860-021-00380-8

**Published:** 2021-08-13

**Authors:** Fatemeh Eini, Maryam Azizi Kutenaei, Fayegheh Zareei, Zeinolabedin Sharifian Dastjerdi, Maryam Hosseinzadeh Shirzeyli, Ensieh Salehi

**Affiliations:** 1 Fertility and Infertility Research Center, Hormozgan University of Medical Sciences, Bandar Abbas, Iran; 2 Infectious and Tropical Diseases Research Center, Hormozgan Health Institute, Hormozgan University of Medical Sciences, Bandar Abbas, Iran; 3 Molecular Medicine Research Center, Hormozgan Health Institute, Hormozgan University of Medical Sciences, Bandar Abbas, Iran; 4 Department of Anatomical Sciences, School of Medicine, Shahid Beheshti University of Medical Sciences, Tehran, Iran

**Keywords:** Bacteriospermia, Leukocytospermia, Sperm DNA integrity, Semen quality

## Abstract

**Background:**

Although bacterial infections have been recognized as a possible cause of male infertility, the effect of bacterial infections on sperm quality and sperm DNA fragmentation remains controversial. The current study aimed to investigate the prevalence rate of bacterial infection in subfertile men and its effect on semen quality. Seminal fluid was collected from 172 male members of infertile couples attending the andrology infertility center and a group of 35 fertile subjects as a control. Sperm parameters and DNA fragmentation were evaluated based on the type of bacteria in all ejaculates.

**Results:**

From the 172 patients investigated for infertility, 60 (34.88%) patients had a positive culture for pathogenic bacteria of different species. Leukocytospermia was significantly higher in infected samples in comparison with non-infected samples (*p* < 0.05). Sperm concentration and motility and morphology were significantly lower in infected than non-infected samples. Moreover, sperm DNA fragmentation was significantly higher in infected than non-infected samples. Besides, our results showed that sperm DNA fragmentation was correlated significantly with leukocytospermia (R: 0.22, *p* < 0.01).

**Conclusion:**

The present study suggested that bacterial infection significantly correlated with leukocytospermia could impair male fertility potential through decreasing sperm motility, morphology, and DNA integrity.

## Introduction

Infertility or subfertility is defined as the failure to achieve a clinical pregnancy after 1 year of regular and unprotected sexual intercourse. A male subfertility is a clinical disorder that accounts for almost 30% of reproductive-aged couples worldwide [1, 2]. The most salient feature of male subfertility is oligoasthenoteratozoospermia. It is characterized by abnormal count, motility, and morphology of spermatozoa in semen samples [3]. Male subfertility could be idiopathic or caused by various factors including immunogenic defects, anatomical and genetic disorders, inflammation reasons, and infection problems [4]. Inflammation pathways could be activated by several inducers including dysfunction in accessory glands, oxidative stress, the anatomical obstacle in the seminal tract, and microorganism infections directly affecting semen quality [5]. Genital inflammation can affect the male reproductive system in various ways. Inflammation directly or indirectly deteriorates spermatogenesis and sperm function through sperm antibody and reactive oxygen species (ROS) production, and DNA fragmentation [6].

Infection is a major cause of inflammation in the male urogenital tract. Untreated-infection may hamper the treatment of infertility relating to the application of assisted reproduction techniques (ART) [7]. Up to 35% of male infertility disorders are associated with urogenital system infections. In this regard, asymptomatic bacteriospermia may be a cause of male-related infertility issues. Bacteriospermia can arise from the male urogenital tract or can be sexually transferred [4].

In the male genitourinary system, infection of the testis, epididymis, and prostate can deteriorate spermatogenesis and fertility potential [8]. Moreover, the infection of the urogenital tract increases the leukocytes in seminal plasma (leukocytospermia) [9]. There are multiple mechanisms to leukocytospermia, such as exposure to environmental toxins, vaginal products during intercourse, alcohol use, tobacco, and certain medications and surgical manipulation [10]. However, it seems there is a significant correlation between bacteriospermia and leukocytospermia in subfertile men.

Bacteriospermia and consequently leukocytospermia can negatively affect male fertility via multiple mechanisms, including involvement in spermatogenesis, deterioration of sperm function, and dysfunction of the genital tract [11]. Bacteriospermia via cellular interactions, sperm adhesion, and agglutination can lead to the impairment of sperm motility [12]. Among pathogenic bacterial species, *E. coli* negatively affects sperm motility [13]. Moreover, leukocytospermia can impact sperm function through the induction of cytokines and ROS generation. In the seminal plasma, the increased ROS level is associated with lipid peroxidation in the sperm plasma membrane and can lead to sperm DNA fragmentation [14]. It has been shown that bacteriospermia and leukocytospermia change the seminal plasma composition, which, in turn, obstructs the genital tract [5]. Moreover, a breach in the blood-testis barrier due to the infection and inflammation pathway causes the formation of an anti-sperm antibody, which may impair sperm function and fertility potential [15].

In the present study, a correlation was found between leukocytospermia and bacteriospermia in asymptomatic men. Moreover, we explored which bacterial species negatively affect sperm function and DNA fragmentation. Therefore, this study aimed to determine the prevalence of bacteriospermia and their impact on semen quality among asymptomatic men in assisted reproduction.

## Materials and methods

### Patients

Semen samples were collected from 172 male partners of the subfertile couples (mean age: 38.2 ± 4.3) attending infertility clinic in a tertiary care hospital between October 2018 and January 2020. A subfertile couple was defined as a couple who failed to achieve a clinical pregnancy after 1 year of regular and unprotected sexual intercourse. These patients had abnormalities in semen analysis results with at least one semen parameter below the reference value recommended by WHO (2010), or with a leukocyte count ≥1 × 10^6^/mL, or displaying any symptom or history of infection in the urogenital system [16]. The patients who were on any antibiotic or surgical therapy in the last month or with anatomical, hormonal, and genetic abnormality were excluded. The 35 fertile men who were attending the clinic during the study were considered as the control group. This study was approved by the ethics committee of the Fertility and Infertility Research and Clinical Center of Hormozgan University of Medical Sciences (# IR.HUMS.REC.1397.208). A signed informed consent form was obtained from all participants.

### Semen collection and analysis

The semen sample was collected at the laboratory after 2–5 days of sexual abstinence. The participants were instructed orally and according to a written protocol to follow a strict procedure: First, they urinated and then washed their hands with a soap. Then, they washed their genital area with antimicrobial soap and rinsed it with physiological saline solution. Finally, the semen obtained by masturbation was collected in a sterile and non-toxic container to be stored in the laboratory.

### Semen preparation

After semen liquefaction (30 min at 37 °C), ejaculates went for a routine andrological analysis, including leukocyte count and semen culture. Volume, pH, concentration, motility, and morphology were analyzed according to the World Health Organization (WHO) guidelines [16].

### Determination of leukocytospermia

Leukocytospermia was performed in accordance with WHO criteria. The presence of polymorphonuclear granulocyte (PMNC) was detected through a histochemical procedure (phloxine–benzidine) detecting peroxidase activity. Leukocytospermia was defined as the presence of > 1 × 10^6^ PMNC/mL of semen.

### Semen bacteriological study and isolation

Immediately after semen collection, all specimens were liquefied at 37 °C for 30–45 min. The ejaculates were aliquot and transferred to a bacteriology laboratory within 3 h for bacteria culture. The ejaculates (100 μL) were cultured on Columbia CNA Agar with 5% of Sheep Blood, MacConkey agar, Thayer-Martin agar, Gardnerella Selective Agar with 5% of Human Blood, Chocolate agar, and Sabouraud agar. The media were incubated for 24 to 48 h in an atmosphere supplemented with 5% CO_2_ at 37 °C to detect aerobic and microaerophilic bacteria. Bacterial identification was carried out biochemically using VITEK 2 system, which provided with colorimetric reagent cards (Gram-positive and Gram-negative cards). The VITEK 2 system was used according to the manufacturer’s instructions with aid of Bergey’s manual of determinative bacteriology [17]. The isolated bacteria in a concentration of > 1 × 10^3^ CFU/mL were considered significant [18].

### Motility assessment

Sperm motility was evaluated under a phase-contrast microscope and 400X magnification. The percentage of motile spermatozoa was evaluated according to WHO guidelines. Sperm motility was assessed on a four-category scheme: rapid progressive, slowly progressive, non-progressive, and immotile. At least 200 sperms in at least five microscope fields of view were counted for each sample.

### Viability assessment

Eosin/Nigrosin staining was used for sperm viability assessment. Staining was performed using eosine-nigrosin (EN comprised of 0.2 g of eosin and 2 g of nigrosin dissolved in a buffered saline solution [153 mM NaCl and 9.65 mM NaH2PO4; pH 7.4]). Equal volumes of sperm samples were then mixed and incubated for 30 s at room temperature. A smear was made on a glass slide and allowed to dry. Unstained or light pink signified live, and red or dark pink colors for the dead were evaluated at 1000X magnification under oil-immersion. Sperm viability was defined as the percentage of live cells. At least 200 sperms were counted for each sample.

### Morphology assessment

Sperm morphology was assessed in semen and the sperm was prepared using the strict criteria. 5 μl of the sample was smeared onto clean glass slides and was allowed to air-dry for 20 mins. The smears were stained by a Diff-quick kit (Baxter Dade diagnostics AG, Dubingen, Switzerland). Morphology assessment was performed according to WHO guidelines (Organization, 2010). To evaluate sperm morphology, at least 200 sperms (100 sperms twice) were counted at 1000X magnification under oil-immersion.

### DNA fragmentation

**Fig. 1:**
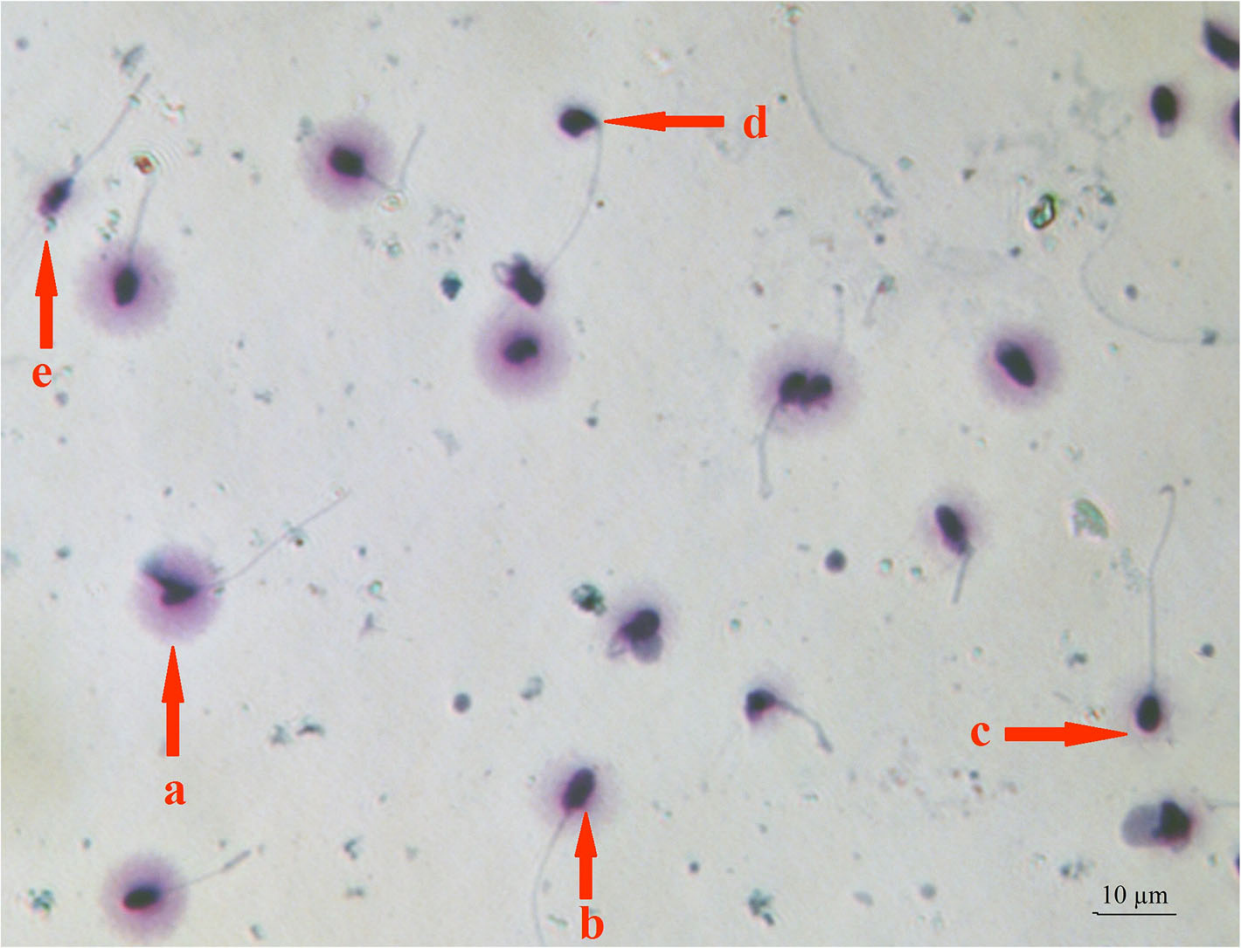
Sperm DNA fragmentation patterns. **a** Big halo. **b** Medium size halo. **c** Small halo. **d** Without halo. **e** Without halo and degraded

Sperm DNA integrity was determined by Sperm DNA fragmentation assay kit (SDFA; ACECR, Tehran, Iran) according to the manufacturer’s instructions [19]. Based on the sperm chromatin dispersion (SCD), five patterns were divided into two groups as follows: sperms with intact DNA: the spermatozoa with large- and medium-sized haloes; sperms with fragmented DNA: the spermatozoa with small-sized haloes or without a hallo or degraded spermatozoa [20]. A minimum of 200 spermatozoa per sample was scored under the light microscopy at 100X magnification. DNA fragmentation was defined as the percentage of spermatozoa with fragmented DNA. Figure 1 shows the 5 patterns of DNA fragmentation.

### Statistical analysis

Statistical analysis was done using the GraphPad Prism data analysis program (GraphPad Software, Inc., San Diego, CA, USA). In the present study, the value of the target variables was represented as mean ± SEM. Results from all sperm parameters were analyzed by either Student’s T-test or one-way ANOVA followed by Tukey’s HSD post hoc test. A simple linear regression (coloration coefficient and R-square) with related curve were used for the evaluation of the relationship between leukocytospermia and sperm DNA fragmentation. Also, Odds ratio (OR) with 95% confidence interval (CI) was used to assess the relationship between leukocytospermia and bacteriospermia. All statistical analyses were two-tailed, and *p* < 0.05 was set as the significance level.

## Results

### Bacterial analysis of semen samples

**Fig. 2:**
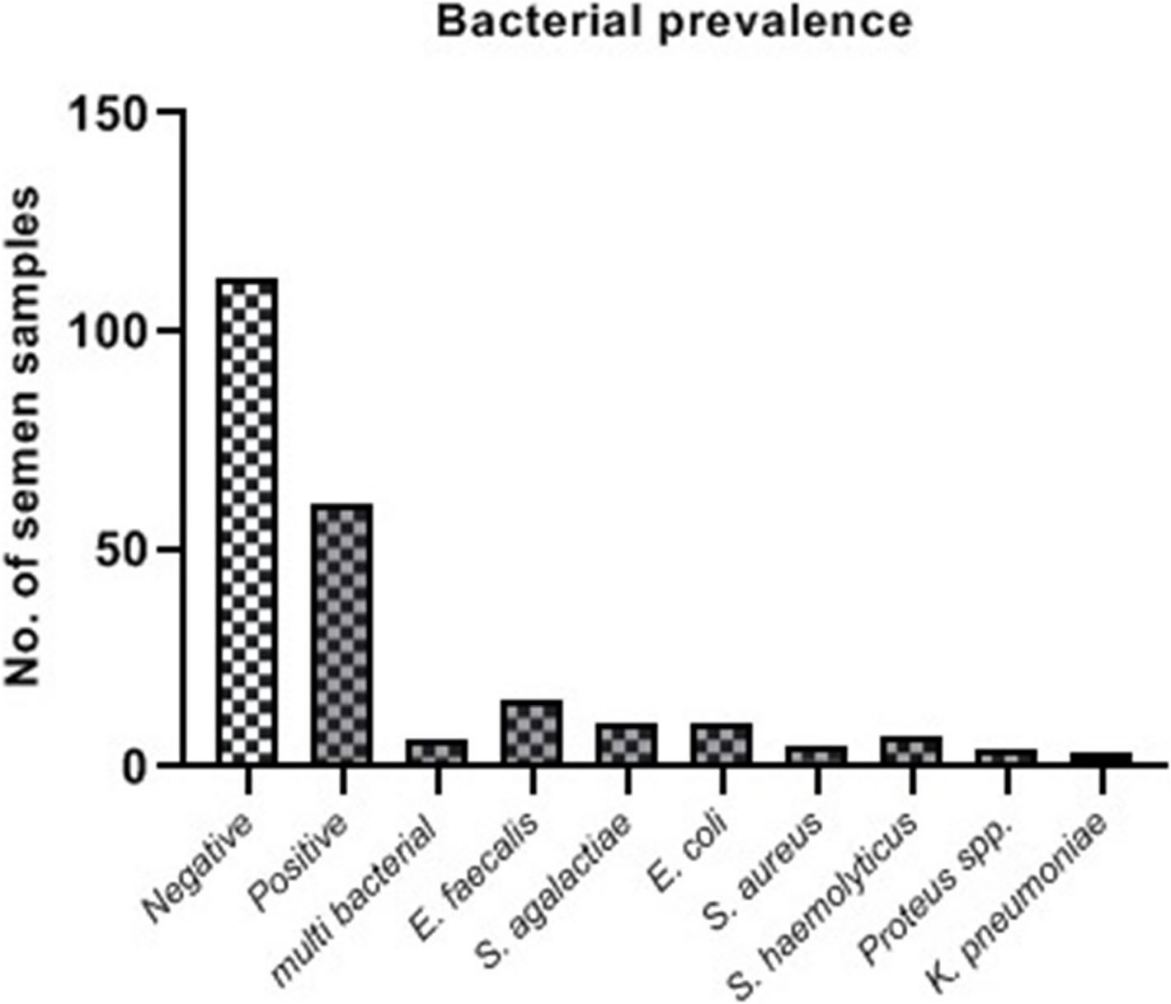
Prevalence of bacterial species in semen samples of subfertile men

Bacterial analysis of semen samples revealed that 112 semen samples (65%) were sterile; 60 cultures (34.88%) were positive with one species, and 6 (3.48%) were infected with more than one species of bacteria in 172 samples. Among positive cultures, *E. faecalis (E. faecalis)* was the most frequent with an occurrence of 25% (15/60). Other frequently recognized bacterial species were *S. agalactiae* (*S. agalactiae)* (16.66%), *E. coli (E. coli)* (16.66%), *S. aureus (S. aureus)* (8.33%), *Staphylococcus (S. haemolyticus)* (11.66%), *Proteus spp*. (6.66%), (*K. pneumoniae) K. pneumoniae* (5%) and multi bacterial (10%). The distribution of isolated bacteria is shown in Fig. 2.

### Effect of bacterial infection on leukocytospermia

The result of leukocytospermia analysis showed that the leukocyte concentration was significantly higher in infected samples in comparison with non-infected fertile and subfertile samples (*p* < 0.01) and (*p* < 0.05), respectively (Table 1). Our results revealed that the association between leukocytospermia and bacterial detection was statistically significant (Odds Ratio: 3.96, 95% CI: 2.04 to 7.68) (Table 2). Also, a significant increase was observed in the leukocytospermia of infected infertile patients regardless of the bacterial infection type (Table 3).

**Table 1:** Comparison of semen and sperm parameters between the healthy fertile men, bacteriospermic and non-bacteriospermic subfertile men

Parameters	Healthy Fertile men	Non-Bacteriospermic subfertile men	Bacteriospermic subfertile men
No.	35	112	60
Age (year)	36.14 ± 18.14	34.24 ± 21.12	35.70 ± 10.83
Volume (mL)	4.05 ± 0.87	4.19 ± 0.90	3.91 ± 1.49
Sperm concentration (10^6^/mL)	75.36 ± 34.12	33.04 ± 11.60 *	24.84 ± 15.93 *
Motility (%)	67.23 ± 20.65	31.71 ± 11.09 *	24.04 ± 11.82 ** #
Progressive motility (%)	39.33 ± 8.06	16.50 ± 7.85 *	11.30 ± 6.80 ** #
Leukocytospermia (10^6^/mL)	1.80 ± 1.90	4.12 ± 3.80 *	5.42 ± 5.49 ** #
DNA Fragmentation (%)	14.33 ± 10.97	23.50 ± 12.81	42.21 ± 19.31 * #
Viability (%)	85.03 ± 13.17	67.67 ± 16.62	55.91 ± 13.56 * #
Normal morphology (%)	6.03 ± 1.05	2.83 ± 1.32 *	1.75 ± 1.13** #

All data are represented as mean ± SEM

* The following values were compared: subfertile men vs healthy fertile men, *p < 0.05, **p < 0.01

# Non-Bacteriospermic subfertile men vs Bacteriospermic subfertile men, #*p* < 0.05

**Table 2:** Correlation of seminal bacterial infection with Leukocytospermia in subfertile males

Bacterial ^a^infection	Semen Sample ^b^			OR (95%CI) ^c^	*P value*
	Leukocytospermia	3.93 (2.04–7.68)	Total		
Positive	38		60	3.93 (2.04–7.68)	*p* < 0.001
Negative	34	78	112		
Total	72	100	172		

^a^ Bacterial infection was explored in a total of 60 semen samples from subfertile men

^b^ Semen samples were considered as leukocytospermia when leukocyte counts were 10^6^/mL

^c^ Odds ratio (OR) with 95% confidence interval (CI) was used to assess relationship between bacterial infection and leukocytospermia in semen samples

**Table 3:** Effect of bacterial infection on sperm parameters in subfertile men

Bacterial species/ semen parameters	Control (35)	*E. Faecalis* *(15)*	*S. agalactiae* *(10)*	*E. coli* *(10)*	*S. haemolyticus* *(7)*	*S. aureus* *(5)*	*Proteus spp.* *(4)*	*K. pneumoniae* *(3)*	Multi bacterial *(6)*
Volume (ml)	4.05 ± 0.87	3.30 ± 1.20	4.02 ± 1.34	2.94 ± 0.89	3.71 ± 2.29	4.80 ± 1.79	3.50 ± 1.91	5.00 ± 1.00	4.00 ± 1.89
Sperm concentration (10^6^/ml)	75.36 ± 34.12	37.17 ± 23.1*	21.00 ± 13.70*	22.33 ± 14.35*	22.14 ± 16.78*	22.80 ± 13.48*	24.25 ± 18.34*	33.33 ± 14.57*	15.83 ± 13.01 **
Viability %	85.03 ± 13.17	52.08 ± 20.1*	52.00 ± 12.90*	54.00 ± 27.71*	58.14 ± 7.17*	52.00 ± 13.73*	64.25 ± 9.84	62.67 ± 5.86	52.17 ± 11.44
Total Motility %	67.23 ± 20.65	29.04 ± 17.3*	29.30 ± 16.92*	19.33 ± 7.91**	16.00 ± 7.87**	20.80 ± 11.21*8	26.50 ± 6.56*	28.00 ± 12.16*	23.33 ± 14.58*
Progressive Motility %	39.33 ± 8.06	16.66 ± 14.8*	12.60 ± 7.47**	8.44 ± 5.17**	7.85 ± 6.25**	10.40 ± 4.16**	11.5 ± 3.70**	9.67 ± 6.81**	13.33 ± 6.06
Morphology %	6.03 ± 1.05	2.33 ± 1.93*	2.10 ± 1.60*	1.34 ± 1.22**	2.07 ± 1.36*	1.60 ± 1.14**	1.25 ± 1.26**	1.33 ± 1.53**	2.00 ± 1.67*
DNA fragmentation %	14.33 ± 18.16	38.04 ± 22.2*	39.00 ± 22.92*	40.00 ± 18.53*	43.14 ± 18.44*	45.00 ± 17.39**	35.00 ± 1.91*	47.00 ± 32.45**	50.50 ± 20.58**
Leukocytospermia %	1.80 ± 1.90	5.21 ± 7.13*	5.70 ± 5.22**	6.56 ± 5.98**	5.28 ± 5.25**	5.40 ± 5.55**	3.25 ± 3.95*	4.33 ± 4.04*	7.66 ± 6.77**

Comparison between different variables in each group with those of the control group (35 fertile individuals). *p < 0.05, **p < 0.01

### Effect of bacterial infection on semen parameters

Table 2 presents the deteriorative effects of bacterial infection deteriorative on sperm parameters in subfertile patients compared to non-infected subfertile and healthy fertile men (control). Sperm viability percentages in infected subfertile patients decreased compared to non-infected and healthy men (*p* < 0.05). The sperm concentration (10^6^/mL) and the percentage of motility and morphology were also significantly lower in infected subfertile samples as compared to non-infected and healthy men (*p* < 0.05 and *p* < 0.01), respectively. Moreover, the results showed that in infected subfertile samples, sperm concentration was decreased more in multi bacterial infection group (*p* < 0.01), as well as the sperm motility in samples with *S. agalactiae*, *E. coli*, *S. haemolyticus*, *S. aureus*, *Proteus spp*., and *K. pneumoniae*. Moreover, morphology percentage was affected more in *E. coli*, *S. aureus*, *Proteus spp*, *K. pneumoniae* (*p* < 0.001) infected groups versus the other infections (Table 3).

### Effect of bacterial infection on sperm DNA fragmentation

**Fig. 3:**
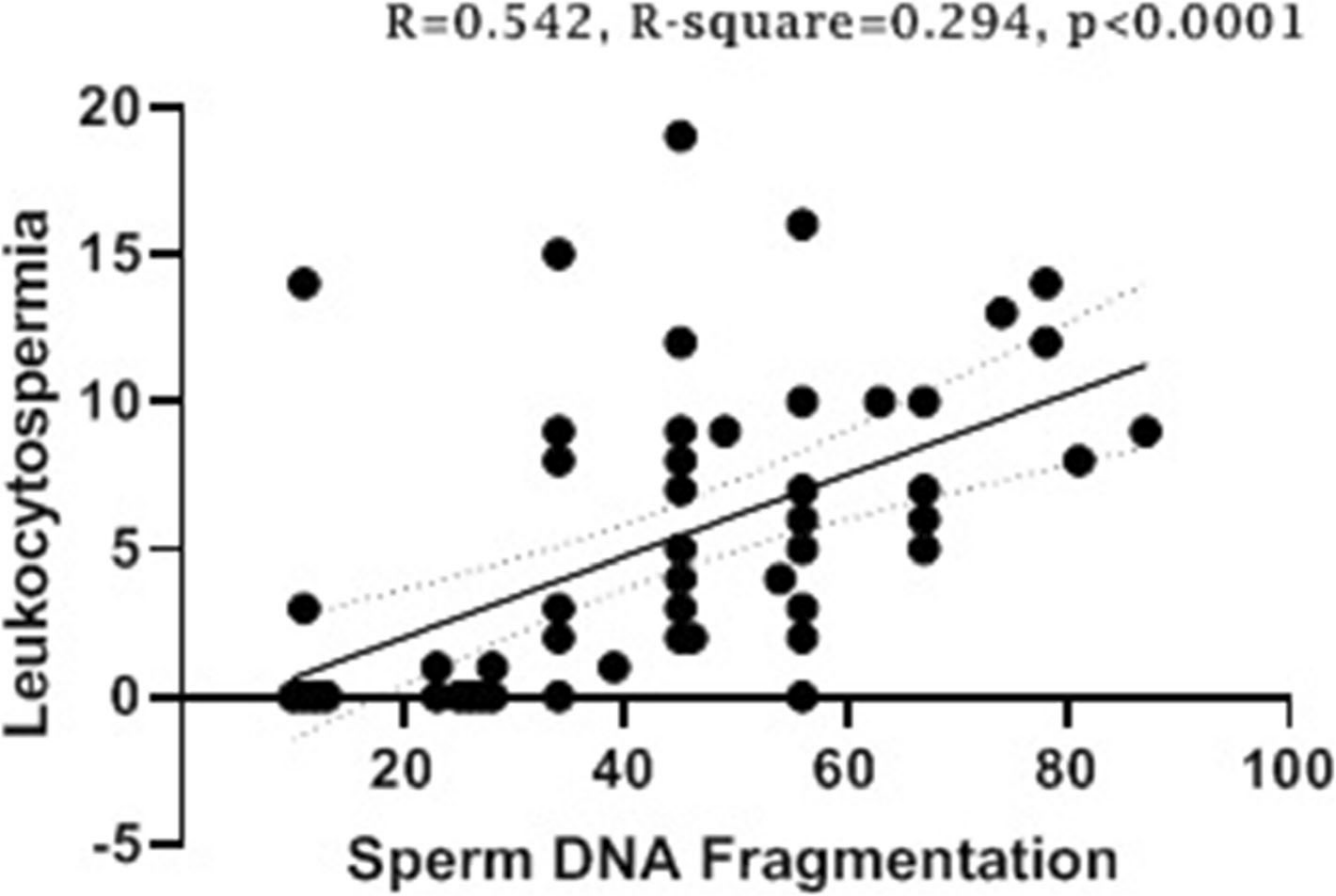
Correlation between leukocytospermia and sperm DNA fragmentation was analyzed by simple linear regression in subfertile semen samples

The result of bacterial infection on sperm DNA fragmentation was shown in Tables 2 & 3. Subfertile infected semen samples had a higher DNA fragmentation than healthy men. Similarly, sperm DNA fragmentation was significantly higher in *S. aureus*, *K. pneumoniae*, and multi bacterial infection groups than other infected (*p* < 0.05) and control groups (*p* < 0.01). Also, a simple linear regression analysis revealed that there is a moderate correlation between leukocytospermia and sperm DNA fragmentation (R-square: 0.23, *p* < 0.0001) (Fig. 3).

## Discussion

Numerous factors can cause male infertility, but the deteriorative effects of bacterial infection on male infertility remain controversial [21]. In the present study, we evaluated the semen quality in subfertile men with at least one abnormality in semen parameters to explore the possible bacterial infection in male infertility. As the results showed, decreases were found in all semen parameters including sperm viability, motility, morphology, and sperm DNA integrity in infected semen. Moreover, these results indicated a statistically significant association between leukocytospermia and bacteriospermia in infected subfertile men. Similarly, a moderate correlation between leukocytospermia and sperm DNA fragmentation revealed that bacterial infection could affect sperm DNA integrity in subfertile men.

Bacterial infection was considered a major cause of infertility in the semen of asymptomatic infertile men [22]. In this study, the bacterial analysis of semen samples from subfertile men showed that 34.88% of samples were infected with one or more than one bacteria species. A similar prevalence rate was detected in the studies by Golshani et al., and Vilvanathan et al. [23, 24]. Other studies reported that the low or high prevalence rate of bacterial infection depended on the human population type [25, 26].. For instance, a low (15%) and a high (46.3%) prevalence rate of bacterial infection have been reported by two researchers, Domes et al. and Ricci et al., respectively [1, 10].. We also showed that the most prevalent bacteria species was related to *E. faecalis*, *S. agalactiae*, *E. coli*, *S. aureus*, *S. haemolyticus*, *Proteus spp.*, and *K. pneumoniae*, respectively. The prevalence rate of bacteriospermia of our results is consistent with Domes et al. with the four most detected species including *E. faecalis* (56%), *E. coli* (16%), group B *Streptococcus* (13%), and *S. aureus* (5%) [10]. *E. faecalis* was more isolated in this study, which is similar to recent studies by Balmelli et al., and Moretti et al. [27, 28].

There is no agreement on how bacteriospermia affects semen parameters, sperm DNA integrity, and ROS production in the semen of subfertile and infertile men. Some studies revealed that bacterial infection has a deteriorative effect on the male urogenital tract [8, 28–30]. However, other studies indicated that bacterial infection could not change the sperm parameters [10, 31]. Among these studies, Hou et al. (2013), in a very small sample size, showed that the type of bacteria did not account for any significant differences in semen parameters of fertile and infertile men. Moreover, Domes et al. showed that the type of bacteria, including *E. faecalis*, *E. coli*, group B *Streptococcus*, and *S. aureus* could not change semen parameters. They showed that sperm motility, morphology, and DNA integrity were not affected by microbes in semen samples [10, 31]. However, our results indicated that the type of bacteria in semen samples influences sperm parameters in subfertile men.

Recent data indicated that both bacteriospermia and leukocytospermia could directly or indirectly affect sperm characteristics depending on the type of bacteria [15]. Domes’ cohort study indicated that leukocytospermia in semen samples, with or without bacteriospermia, had a deteriorative effect on semen parameters, including sperm concentration, motility, and morphology [10]. Leukocytospermia is an inflammatory condition possibly attributed to inflammation or infection in the semen [32]. Although many factors are associated with leukocytospermia, we found that leukocytospermia was correlated with bacterial infection (*p* < 0.001) regardless of the type of infection. This was evident from the fact that 38 samples were found with bacteriospermia among 72 leukocytospermia positive semen samples, suggesting that leukocytospermia could be a predictor of bacterial infection in subfertile men. This is because potential pathogens in the genital tract lead to an inflammatory process with an increase of leukocytes in the seminal fluid [33]. In this regard, Feraczek et al. (2014) suggested that microbial detection should be recommended in semen samples with leukocytospermia, especially in subfertile and infertile men. A number of researchers showed that there is no statistically significant relationship between leukocytospermia and bacteriospermia in the ejaculated semen [15]. This controversy may be due to the elimination of bacteria in the urogenital tract by leukocytes in the final stage of the inflammatory process.

Regardless of leukocytospermia, many studies showed that bacterial contamination could directly deteriorate sperm quality to a great extent in the semen analysis. For instance, the interactions between glycoproteins and receptors in the sperm surface and bacterial flagella could cause sperm agglutination and some adhesion occurrences indicated sperm motility loss [28]. Furthermore, *E. coli* and *Staphylococcus* were found to have an adhesion with receptors on male gametes [15]. Besides, soluble spermatotoxic factors such as sperm immobilization factor alter sperm viability and motility by reducing mitochondrial ATPase functionality and membrane potential [7]. Consistent with the previous findings, our results showed that sperm viability was significantly reduced in infected semen compared to the non-infected. In this regard, Varela et al. showed that viable sperms were significantly decreased in a load of microbial contamination in semen samples [34].

Sperm concentration is another semen parameter which plays a crucial role in male infertility [35]. Our results also showed that sperm concentration was decreased in infected semen in comparison with non-infected samples. This finding was consistent with the other studies showing the probable causative role of bacteriospermia. Consequently, the leukocytospermia of infected samples negatively affects sperm concentration [36]. In the present study, the increased leukocyte count in semen samples is associated with a lower sperm concentration. Our results are consistent with a number of studies indicating that both bacteriospermia and leukocytospermia reduced sperm concentration in the infected samples with *E. faecalis* and *E. coli* negatively affected sperm concentration [7, 28]. In this regard, Pajovic et al. confirmed that sperm concentration was increased after antibiotic therapy in infected semen or pyospermia ejaculates [37]. Similarly, the positive effect of antibiotic treatment on sperm concentration was proven by Ahmadi et al. in the infected semen with *M. genitalium* infection [22].

Sperm morphology and motility have proved to be remarkable factors in semen parameters [35]. Sperm morphology abnormalities have been observed in patients with semen or urogenital tract infections. These abnormalities include elongation and reduced acrosomal inducibility, tapering of sperm head and neck, and anomalies of the sperm tail [38]. It has been shown that poor sperm morphology is usually associated with sperm nuclear defects caused by inflammatory or infectious urogenital tract. Studies showed that in semen contamination samples, altered sperm morphology was attributed to bacterial infection and leukocytospermia. In these samples, the evaluation of sperm nuclear defects also showed poor sperm morphology, especially in the sperm head accompanied by the sperm DNA integrity [15]. In the present research, poor sperm morphology was detected in all infected samples in comparison with the non-infected. These findings are consistent with Mehta et al. (2002), and Villegas et al. (2005) that reported several types of bacteria like *E. coli* and *E. faecalis* to adversely affect sperm morphology [39, 40]. Besides, Zeyad et al. (2017) noticed an increase of abnormal morphology in the infected semen samples [7]. The direct contact between attachment organelles of bacteria and spermatozoa was found for *E. coli* and *S. haemolyticus*. These contacts between bacteria and spermatozoa directly immobilize spermatozoa and affect its motility and morphology via bacterial pili or fimbriae and mannose receptor-dependent interactions [7]. We also showed that sperm motility was significantly reduced in contaminated semen samples compared with the non-infected. Based on functional studies, several bacterial components such as immune-dominant antigen A, alkaline shock proteins, and thermonuclease can have deteriorative effects on sperm motility. These ingredients are introduced as bacterial virulence factors, and antibacterial resistance was found in the cell wall or culture supernatants. According to Li et al. (2018), *S. aureus*, through virulence components, damages sperm motility, and morphology [41]. In a similar vein, our results showed a loss of motility in seminal bacterial contamination, especially for *E. coli*, *S. aureus,* and *S. haemolyticus.*

Several studies indicated that sperm DNA integrity is considered an essential male factor in successful natural pregnancies [6, 7]. It seems that sperm DNA condensation plays an inevitable role in male fertility, and early embryonic development is affected by sperm DNA integrity [21]. Earlier studies revealed that both leukocytospermia and excessive ROS production following bacteriospermia lead to sperm DNA fragmentation [14]. In fact, during bacterial infection, seminal ROS level might be increased in patients with urogenital tracts infection [42]. The influence of bacteria on semen parameters may be due to its ability to produce some inflammatory mediators and leukocytes recruitment and consequently ROS elevation. Elevated ROS levels along with leukocytospermia has a deteriorative effect on sperm DNA fragmentation [15].

The present findings showed that sperm DNA fragmentation was significantly higher in infected patients than the non-infected. Moreover, we found a statistically significant correlation between leukocytospermia and sperm DNA fragmentation. Zeyad et al. (2017) reported that bacterial infection has a negative impact on sperm DNA condensation [7]. A similar result was reported by Rybar et al. (2012) with different species of bacteria. These researchers also reported that after antibiotic treatment, the DNA fragmentation was significantly decreased in patients with urogenital infection [4]. Moreover, Domes et al. (2012) indicated a significant correlation between DNA fragmentations and leukocytospermia, which is consistent with the present findings [10]. Altogether, it seems that bacterial infection via stimulation of inflammatory mediators can affect DNA integrity in patients with seminal infection.

Our results principally are consistent with the previous studies of the effect of bacterial infection on sperm parameters. Despite most studies indicating no correlation between leukocytospermia and bacteriospermia, our results revealed that leukocytospermia was significantly associated with bacteriospermia. Moreover, a moderate correlation was observed between leukocytospermia and sperm DNA fragmentation in subfertile men.

Still, some limitations can be considered in this study. Even though the sample collection procedure followed WHO protocol, some skin bacteria from the hands or penile skin flora could contaminate semen samples. Moreover, bacterial species like *C. trachomatis*, Mycoplasma species, and Ureaplasma species are not detectable by the routine bacterial culture method. Therefore, the false-negative culture is not inevitable. It could occur in the samples with leukocytospermia and without bacteriospermia. However, the main limitation of this study was the small number of samples with low quantities of semen. It limited us to have additional experiments like ROS-level examination and polymerase chain reaction (PCR). Therefore, future studies with larger samples and higher amounts of semen for ROS level examination and PCR will better consider the effect of the semen microbiota on sperm DNA fragmentation and oxidative status in subfertile men semen.

## Conclusion

Many specific factors can lead to male infertility. This study suggested that leukocytospermia could be a predictor of seminal bacterial contamination in subfertile men. Our results revealed that bacterial infection has deteriorative effects on semen parameters. Thus, leukocytospermia and bacteriospermia might affect sperm DNA integrity and decrease embryonic development and pregnancy outcomes. Therefore, it seems that urogenital tract infection treatment is valuable to improve the pregnancy rate in subfertile men. Further investigations of this topic are required.

## Data Availability

The datasets used and/or analyzed during the current study are available from the corresponding author upon reasonable request.
